# Effective Voriconazole in an Immunocompetent Patient With Amphotericin B Resistant Systemic Cryptococcal Granulomatosis

**DOI:** 10.7759/cureus.11101

**Published:** 2020-10-23

**Authors:** Khalid Serraj, Habiba Alaoui, Ahmed Amine El Oumri, Mohamed Barrimi, Houda Bachir

**Affiliations:** 1 Internal Medicine, Faculty of Medicine and Pharmacy, Mohammed Premier University, Oujda, MAR; 2 Immunohematology Cellular Therapy, Faculty of Medicine and Pharmacy, Mohammed Premier University, Oujda, MAR

**Keywords:** systemic, granulomatosis, cryptococcosis, immunocompetent, resistance, voriconazole, monotherapy

## Abstract

The diagnostic management of systemic granulomatosis is a difficult clinical exercise. The most frequent etiologies are tuberculosis and sarcoidosis. However, it is important to search as well for the other causes of granulomas, especially infections and malignancies, the prognosis of which can be poor without adequate treatment. A 67-year-old immunocompetent patient presented with granulomatous adenitis without caseous necrosis. The etiological evaluation had revealed neurological, pulmonary and lymph node systemic cryptococcosis. Conventional antifungal therapy with the triple combination Amphotericin B - Flucytosine - Fluconazole has not been effective, indicating administration of voriconazole. The evolution was rapidly favorable with apyrexia after 48 hours, disappearance of clinical symptoms, normalization of biological parameters of cerebrospinal fluid (CSF) and major improvement of radiological abnormalities. This clinical case is original by the disseminated involvement, the patient's non-immunocompromised status and the primary resistance to amphotericin B. Our findings underline the importance of carrying out an exhaustive evaluation, reflecting on cryptococcosis in any systemic granulomatosis and knowing the various therapeutic alternatives, in particular, voriconazole if primary response to amphotericin B has not been obtained.

## Introduction

Systemic cryptococcosis is a rare but also underdiagnosed condition because of its often-confusing clinical presentation. It typically occurs in immunocompromised, especially HIV-patients. The treatment is based usually on Amphotericin B - Flucytosine - Fluconazole and is effective in almost all cases [1]. We present a rare observation of systemic neuromeningeal, pulmonary and lymph node cryptococcosis of an immunocompetent, which has been shown to be refractory to usual first-line antifungal therapies.

## Case presentation

A 67-year-old patient, with no particular pathological history, was admitted to the Department of Internal Medicine with severe asthenia and fever varying between 38 and 38.5°C. These symptoms had been present for three months before admission and had motivated several outpatient consultations. There was no history of associated neurological, respiratory or digestive signs

**Figure 1:**
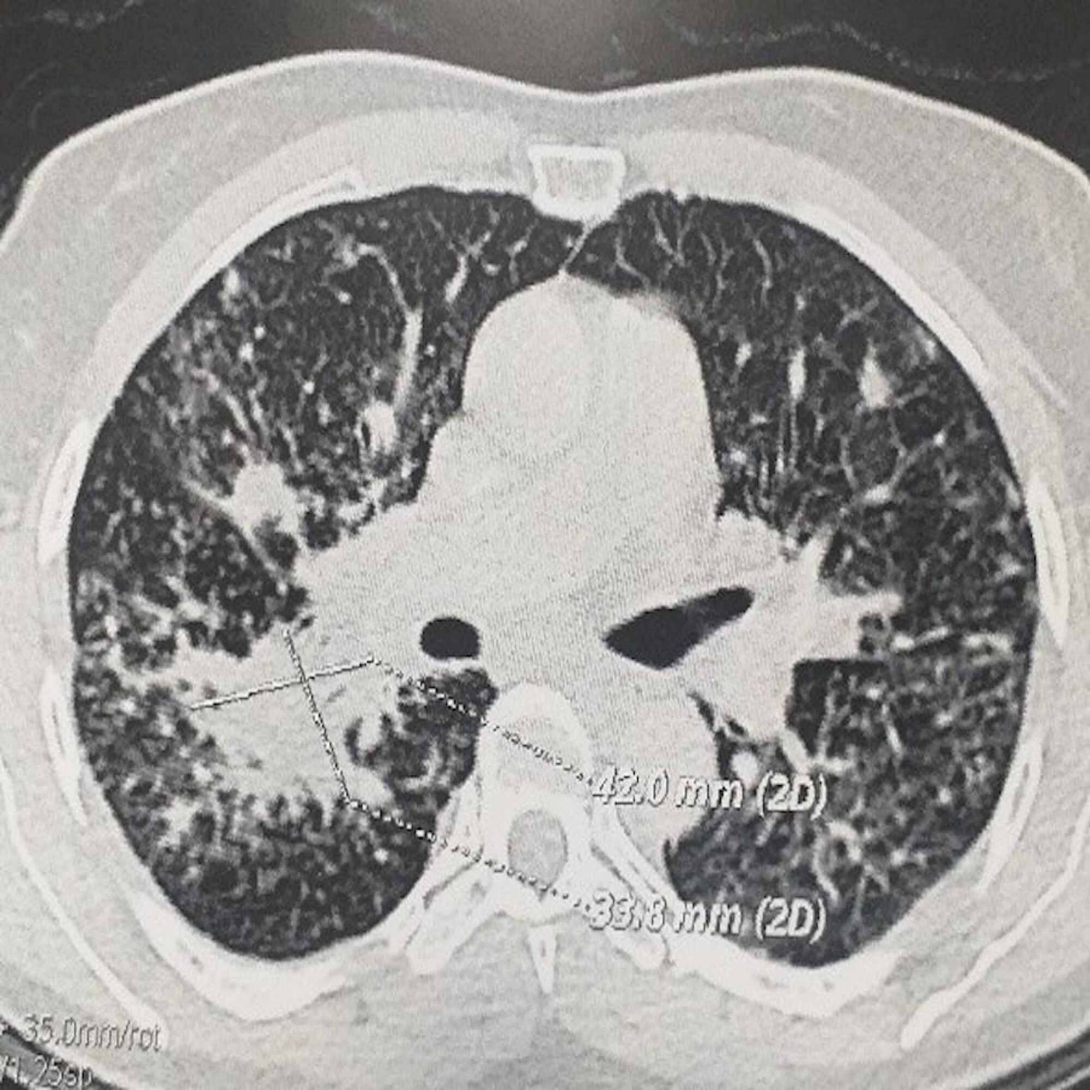
Chest CT scan

**Figure 2:**
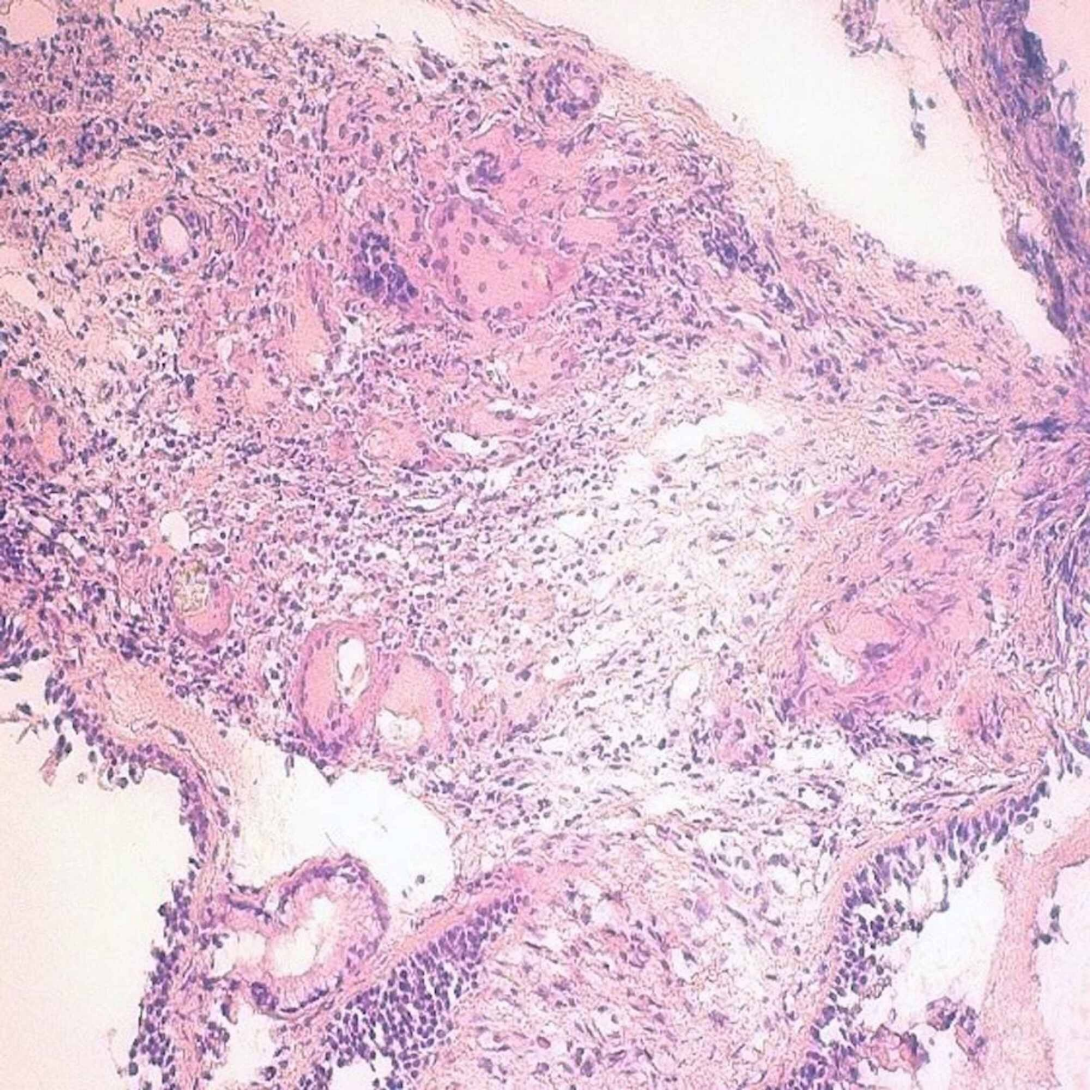
Lymph node granuloma (haematoxylin and eosin stain; original magnification × 100)

**Figure 3:**
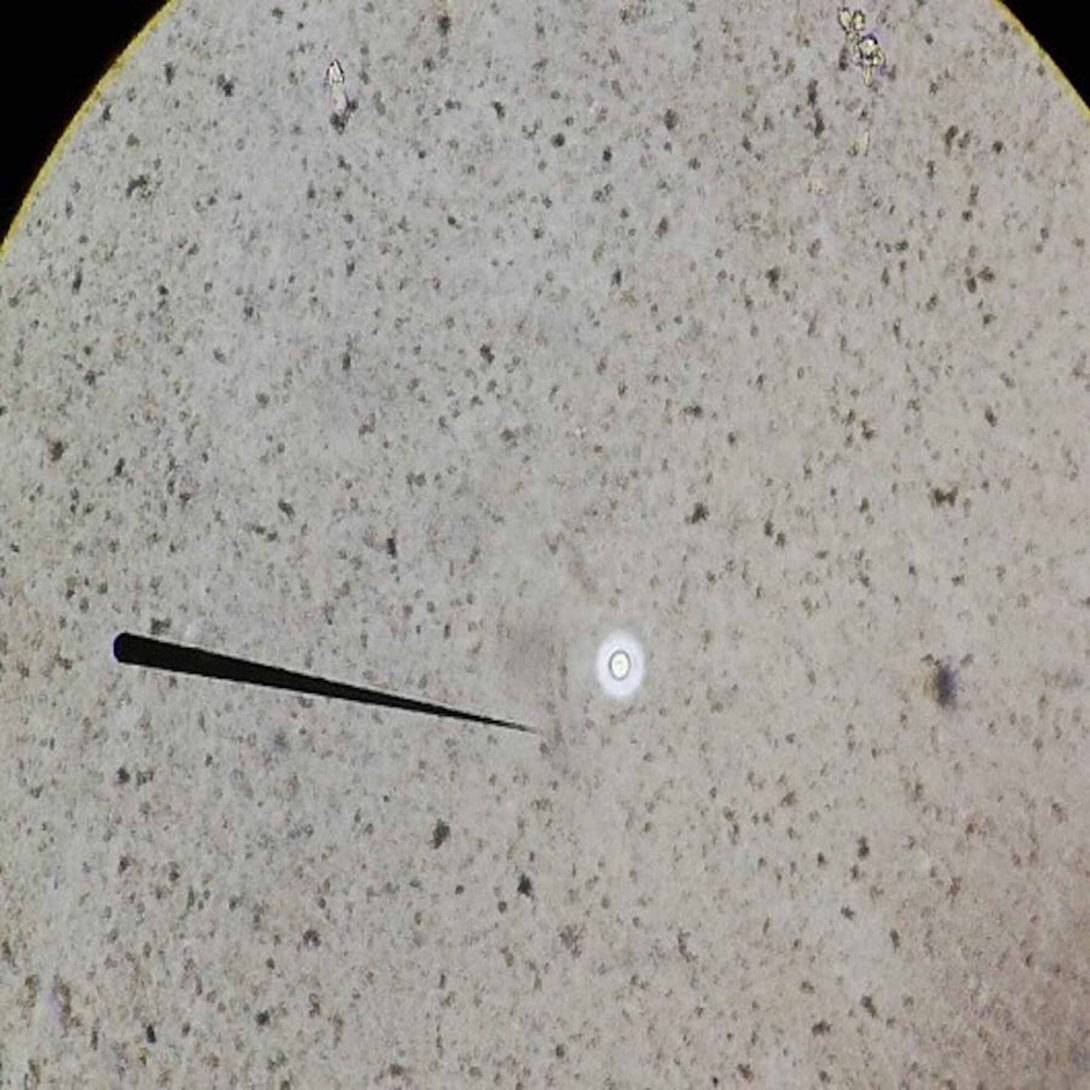
Positive research for cryptococcus in CSF (Chinese ink staining) CSF, cerebrospinal fluid.

Laboratory tests revealed a normochromic microcytic anemia at 9.6 g/dl and lymphopenia at 850/mm^3^ the hemogram, a serum ferritin level at 160 ng/ml, a C-reactive protein at 25 mg/l and an inflammatory profile at the electrophoresis of serum proteins. The chest X-ray, abdominal ultrasound, cytobacteriological examination of the urine and blood cultures performed at the time of feverish peaks did not reveal any particular abnormalities. HIV serology, anti-nuclear antibodies and Quantiferon-TB were negative. Cervico-thoraco-abdomino-pelvic CT scan revealed the presence of multiple deep cervico-mediastinal lymphadenopathy measuring between 8 mm and 25 mm, a significant interstitial syndrome and parenchymal condensations reaching more than 4 cm (Figure 1). Due to the absence of an obvious hematological cause of the polyadenopathy, a biopsy by mediastinoscopy had shown the presence of specific granulomatous adenitis without caseous necrosis or tumor elements. An immunohistochemical supplement had subsequently confirmed this result. Bronchoscopy with biopsy and broncho-alveolar lavage showed granulomatous inflammation with negative microbiological analysis (Figure 2). The sarcoidosis hypothesis was initially raised, but it was more appropriate to look for other granulomatosis, notably tuberculosis because of the endemic nature of the latter in our Moroccan context. A search for BK in the sputum was therefore carried out and was negative three times. No toxic or medicinal intake was suspected and clinical data as well as serologies of leishmaniasis, brucellosis, cytomegalovirus, toxoplasmosis and borreliosis made it possible to exclude an active granulomatous infection. In the doubt of a tuberculosis, a quadrilateral anti-bacillary test based on rifampicin, isoniazid, pyrazinamide and ethambutol was started and the patient was readmitted 10 days after for fever 38.3°C, extreme fatigue, severe headache and dyspepsia. An emergency hospitalization was therefore organized with a biological assessment, which made it possible to eliminate any possible toxicity of anti-tuberculosis drugs. Cerebral CT was normal and the lumbar puncture had shown a clear cerebrospinal fluid (CSF) with 12 lymphocytes/mm^3^, and normal glucose and protein levels. The direct bacteriological examination and the cultures were negative but the Chinese ink direct examination revealed the presence of cryptococci thus allowing to retain the diagnosis of neuro-meningeal cryptococcosis (Figure 3). The soluble Cryptococcus antigens were also positive. A three-week treatment with amphotericin B at a dose of 0.7 mg/kg/day IV in combination with flucytosine at a dose of 25 mg/kg/6 h was started with a relay with fluconazole at a dose of 400 mg/day. The outcome had shown only a partial improvement on systemic symptoms as well as headaches most probably due to iterative decompressive lumbar punctures. The control CSF study, on the other hand, showed a progressive increase in the cellularity of the CSF under treatment with leukocytes having reached 52/mm^3^ after one month of treatment and a persistence in the positivity of soluble antigens.

Otherwise, the immunosuppression assessment other than HIV was normal, namely the serum protein electrophoresis, the determination of the various fractions of the complement, the blood lymphocyte immunophenotyping, as well as the immunoglobulin fractions weight test. There was no diabetes mellitus.

**Figure 4:**
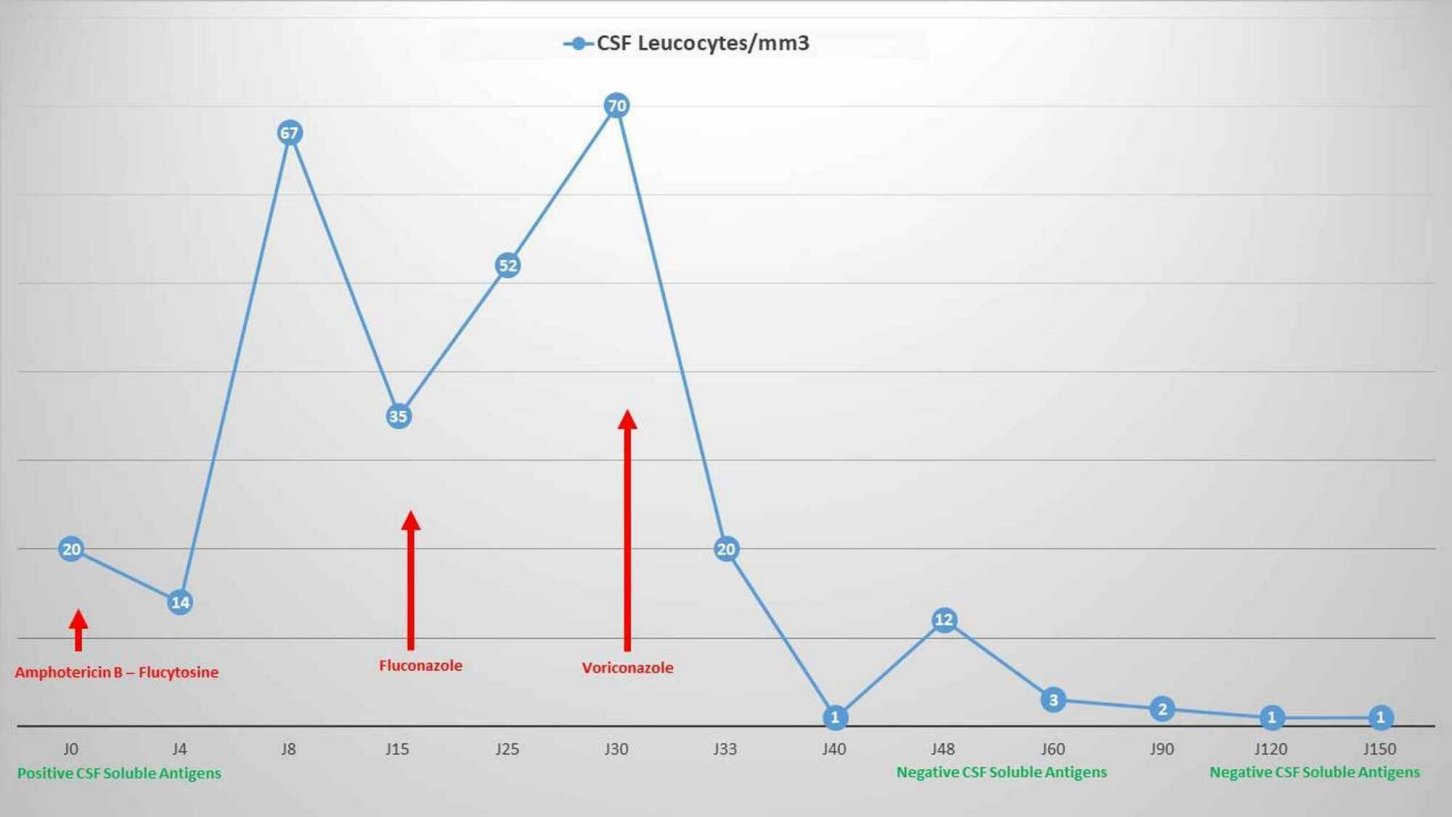
Evolution of CSF cellularity and soluble antigens according to treatment CSF, cerebrospinal fluid.

The appearance of recurrent convulsive states, the worsening of the spinal cell had pushed to rule out the other possible differential diagnoses of meningitis with clear liquid in particular, tuberculosis, herpetic meningitis, listeriosis, inflammatory meningitis and neoplastic meningitis was ruled out and the initial antifungal treatment was replaced by voriconazole at a dose of 400 mg/day IV and then orally for three months, followed by maintenance at a dose of 200 mg/day still in progress. The clinical course was quickly favorable in less than 48 hours with the achievement of apyrexia, complete disappearance of the headaches and resumption of progressive activity by the patient. Close checks of the CSF had shown negativation of soluble antigens as well as a gradual decline in CSF cellularity that normalized completely after one month (Figure 4). In addition, the control CT had shown three months after the start of treatment with voriconazole a few infra-centimeter lymph nodes of residual appearance. The parenchymal images also observed previously had also completely disappeared (Figure 1).

## Discussion

Cryptococcosis is a fungal infection transmitted by inhalation. The lung therefore constitutes the main gateway before subsequent dissemination to other organs. Cryptococcus Neoformans and Cryptococcus Gattii are the two most involved agents. Mortality during cryptococcal infection is on average 25% but can exceed 60% in patients with neurologic involvement. Frequent delayed diagnosis and emergence in immune-compromised patients are the two main explanations for this high mortality [1]. The incidence of cryptococcosis has increased considerably since the 1970s due to the arrival of immunosuppressive and anticancer therapies, organ and tissue transplants and HIV infection. In immunocompetent people, systemic cryptococcosis is rare [2,3]. In our patient, an exhaustive immunological assessment had not revealed immunosuppression. We believe important however to continue to monitor patients closely as the fungal infection may be indicative of latent immunosuppression which can only become symptomatic in the future.

Cryptococcal infection affects lungs, nervous system, skin, and much less frequently the eye, the digestive tract, the urogenital sphere and the musculoskeletal system. Lymph node involvement has been exceptionally described but finds its full meaning in the granulomatous nature of the anti-cryptococcal immune response. In our patient, the first revealing manifestation was the general signs associated with a deep chest polyadenopathy which turned out to be granulomatous on the biopsy data and without respiratory or neuro-meningeal symptoms at the initial phase. This observation underlines the importance of a broad and systematic etiological exploration of all the causes of granuloma before retaining the diagnosis of sarcoidosis whose treatment, when indicated, is mainly based on corticosteroids and immunosuppressive therapy [1,4]. Table 1 summarizes the main causes of systemic granulomatosis.

**Table 1:** Etiologies of granuloma

Medicines	Infections	Dysimmunity	Malignancy
Interferon α	Mycobacteria	Crohn Disease	Lymphoma
Anti-TNF α	Brucella	Lupus	Lung
Statins	Listeria	Rheumatoid Arthritis	Breast
Fibrates	Syphilis	Sarcoidosis	Breast
Isoniazide	Tularemia		Prostate
Quinine	Whipple Disease		Cervix
Allopurinol	Nocardia		
Beryllium	Borrelia		
Cerium	Cytomegalovirus		
	Epstein-Barr virus		
	Leishmania		
	Toxocara		
	Toxoplasma		
	Bilharzia		
	Histoplasma		
	Candida		
	Aspergillus		
	Cryptococcus		

The treatment of crytococcal infection usually takes place in 2 to 3 phases: induction, consolidation and maintenance. The overall duration of treatment is about one year and/or until the eventual disappearance of the underlying immunosuppression. In the case of the immunocompetent patient, this duration can be revised downwards to between 6 months and one year, with close monitoring thereafter. The three molecules of choice are amphotericin B, flucytosine and fluconazole [1,5]. The prevalence of relapses after treatment with Amphoteric B - Fluconazole is around 23%, mainly related to poorly managed maintenance treatment. Cases of primary resistance are rather rare and mainly linked to errors concerning the therapeutic modalities. In our patient, treatment with amphotericin B and fluconazole was done according to the recommendations, which presupposes the presence of intrinsic resistance. Theoretically, amphotericin remains the antifungal of choice because the most powerful and having the most powerful fungicidal activity in vitro in comparison with voriconazole whose effect is essentially fungistatic, particularly in CSF. In real life, the resistance of cryptococcus to amphotericin B in the immunocompetent is exceptional [6-8].

In most cases of cryptococcosis reported in the literature, voriconazole was associated with moderate doses of amphotericin, unlike our observation that it was monotherapy for the entire duration of treatment. This efficacy is explained by the activity of voriconazole but also by its significant systemic diffusion including the CSF. Other antifungals that have shown some efficacy in cryptococcosis are itraconazole, posaconazole and isavuconazole (Table 2) [9]. In our context, the ideal would have been to test the antifungal activity of different drugs in order to provide documentation of the resistance, but we have opted to switch quickly to voriconazole due to the vital emergency and acute encephalitic complications.

**Table 2:** Therapeutical options against cryptococcus

Clinical situation	Induction	Consolidation	Maintenance
Non-HIV patients	Amphotericin B 0,7 mg/kg/day IV + Flucytosin 25 mg/kg/6 h orally 2-4 weeks	Fluconazole 400 mg/day orally 8 weeks	Fluconazole 200 mg/day 6-12 months
HIV patients	Liposomal Amphotéricin B 3-4 mg/kg/day IV + Flucytosin 25 mg/kg/6 h orally 2-4 weeks	Fluconazole 400 mg/day orally 10 weeks	Fluconazole 200 mg/day 6-12 months
Amphotericin-resistant patients	Voriconazole 400-800 mg/day IV orally Or Itraconazole 400 mg/day orally 3 months		Voriconazole 200-400 mg/day orally Or Itraconazole 200 mg/day orally 6 to 12 months

## Conclusions

The multi-systemic neuromeningeal, respiratory and lymph node nature of infection, the non-immunocompromised status and the initial resistance to amphotericin B make, in our knowledge, of this observation a unique case. Several lessons can be learned, in particular the importance of making an exhaustive assessment in the face of any systemic granulomatosis, to think of cryptococcosis even if the patient is not immunocompromised and finally to know the different therapeutic alternatives, in particular voriconazole in case of primary non-response to amphotericin B.
